# Targeting Oxidatively Induced DNA Damage Response in Cancer: Opportunities for Novel Cancer Therapies

**DOI:** 10.1155/2018/2389523

**Published:** 2018-03-27

**Authors:** Pierpaola Davalli, Gaetano Marverti, Angela Lauriola, Domenico D'Arca

**Affiliations:** ^1^Department of Biomedical, Metabolic and Neural Sciences, University of Modena and Reggio Emilia, 41125 Modena, Italy; ^2^Department of Life Sciences, University of Modena and Reggio Emilia, 41125 Modena, Italy; ^3^Istituto Nazionale di Biostrutture e Biosistemi, 00136 Roma, Italy

## Abstract

Cancer is a death cause in economically developed countries that results growing also in developing countries. Improved outcome through targeted interventions faces the scarce selectivity of the therapies and the development of resistance to them that compromise the therapeutic effects. Genomic instability is a typical cancer hallmark due to DNA damage by genetic mutations, reactive oxygen and nitrogen species, ionizing radiation, and chemotherapeutic agents. DNA lesions can induce and/or support various diseases, including cancer. The DNA damage response (DDR) is a crucial signaling-transduction network that promotes cell cycle arrest or cell death to repair DNA lesions. DDR dysregulation favors tumor growth as downregulated or defective DDR generates genomic instability, while upregulated DDR may confer treatment resistance. Redox homeostasis deeply and capillary affects DDR as ROS activate/inhibit proteins and enzymes integral to DDR both in healthy and cancer cells, although by different routes. DDR regulation through modulating ROS homeostasis is under investigation as anticancer opportunity, also in combination with other treatments since ROS affect DDR differently in the patients during cancer development and treatment. Here, we highlight ROS-sensitive proteins whose regulation in oxidatively induced DDR might allow for selective strategies against cancer that are better tailored to the patients.

## 1. Introduction

Human cancer is the primary death cause in economically developed countries and the second death cause in developing countries. Adoption of cancer-associated lifestyles as smoking, physical inactivity, and “westernized” diets and the increasing number of aging people are major causes for cancer expansion [1]. Targeted therapy has improved the outcome for specific cancer types; however, intrinsic or acquired resistance to the therapies remains an inevitable challenge for the patients [2–4]. Several features like cell composition of the tumor, tumor microenvironment, and drug efficiency lead tumor cells to overwhelm the therapies through the same mechanisms that healthy cells utilize for surviving under adverse conditions. In addition, many therapies are scarcely selective for cancer cells and damage healthy cells thus compromising the therapeutic effect [5–7]. Almost all human tumors are characterized by genomic instability, which essentially derives from deoxyribonucleic acid (DNA) damage generated by reactive oxygen/nitrogen species (ROS/RNS, usually referred as ROS), ionizing radiation, and chemotherapeutic agents, besides occasional genetic mutations, so that DNA damage is direct and indirect target of a wide number of anticancer treatments [8–11]. Eukaryotic cells have developed a sophisticated signaling-transduction mechanism, named DNA damage response (DDR), that maintains cell genome integrity by acting as an efficacious network. DDR can detect DNA lesions and arrest the cell cycle both temporary (checkpoint control activation) and permanently (senescence) or promote cell death (apoptosis). DDR sets cell fate depending on mode and level of DNA damage after comparing its severity and cell potentiality to survive. Aberrant repair mechanisms, mutations, and polymorphisms of genes involved in DNA repair contribute to human cancer onset, development, and progression [12–15]. DDR defects that are detectable in human tumors allow classifying the patients for appropriate therapy. Tumor cells often shift their ratio between DNA damage and DNA repair activities in favor of repair that leads to stabilize DNA lesions, as the repairing system cannot identify gene mutations. The lesion extent may exceed the repairing capability of the cell and generate resistance to DNA-targeted therapies [16–18]. Mechanism-based-targeted therapies are preferentially administered as single-target therapies often induce resistance through restoring basal cancer pathways [19–21]. Oxidatively induced DDR has aroused increasing interest since when ROS are no more considered causing exclusive molecular damage or palliative effect against anticancer drugs. ROS together with related molecules and enzymes contribute to physiological functions and pathological alterations of DDR. Oscillations of the redox equilibrium under the cell death threshold can affect the stringency of DDR through modulating its pathways and mechanisms [22–24]. ROS participate to the complex crosstalk of DDR and autophagy that contributes to treatment resistance of cancer cells and their subsequent regrowth through the DNA repair mechanisms [25–29]. Depending on their level, ROS coordinate intracellular redox signaling by acting as messengers in both healthy and cancer cells, although through different pathways. The imbalance between ROS/RNS production and elimination favors their accumulation, subjecting both healthy and cancerous cells to the oxidative/nitrosative stress (collectively named oxidative stress, OS). Cancer cells proliferate in a constitutive OS state, as their hallmark, that may generate resistance to ROS-based anticancer interventions when the antioxidant system of the cell is proportional to its OS level or evolve towards cell death when ROS are subjected to spontaneous or therapeutically induced further increase [30–35].

Here, we briefly prospect possible points of therapeutic intervention in oxidatively induced DDR regarding ROS homeostasis involvement that are under investigation as mechanism-based therapeutic strategies to counteract the human cancer.

## 2. ROS Homeostasis

### 2.1. Production of ROS and RNS

The oxidative metabolism in mitochondria constantly produces a flux of reactive oxygen species (ROS) and a flux of reactive nitrogen species (RNS) as oxidative phosphorylation by-products. The production is estimated on average 1-2% of total rate of oxygen consumption in healthy human body. ROS/RNS are usually named free radicals since they are the most important classes of the free radical family in the majority of living organisms. Free radicals contain an atom or a molecule with one or more unpaired electrons that make them highly reactive, able to bind other radicals or oxidize molecules that they contact. Free radicals share a short life and a generation of chain reactions that ultimately lead to cell structure damage. ROS comprise the singlet oxygen (½ O), the superoxide anion radical (O_2_⨪) and its metabolites, as the very toxic hydroxyl radical (^•^OH), and the nonradical hydrogen peroxide (H_2_O_2_) that, in the presence of redox active metals, is partially reduced to (^•^OH), by Fenton reaction [36]. The mitochondrial respiratory chain leaks electrons causing partial oxygen reduction to O_2_⨪, which is spontaneously, or by superoxide dismutase2 (SOD2), rapidly transformed into H_2_O_2_. Also, peroxisomal NADPH oxidases (NOXs) are implicated in electron transfer from intracellular NADPH to oxygen generating O_2_⨪ that is converted into H_2_O_2_ by superoxide dismutase3 (SOD3). The overall H_2_O_2_ is turned into reactive ^•^OH radicals. RNS were derived from the very dangerous peroxynitrite (ONOO−) generated by O_2_⨪ and nitric oxide (^•^NO), a highly reactive gaseous molecule, but not a radical, soluble in water and diffusible across cell membranes. The reaction is catalyzed by NO synthases (NOS1–3), a family of constitutive or inducible enzymes with different tissue distribution utilizing arginine and NADPH. ^•^NO competes with SOD by directing O_2_⨪ towards ONOO−, rather than H_2_O_2_. NO-derived oxidants are endowed with cellular antimicrobial action and act with ROS contributing to establish oxidative conditions [37, 38].

### 2.2. Antioxidants (ROS Scavenging System)

**Figure 1:**
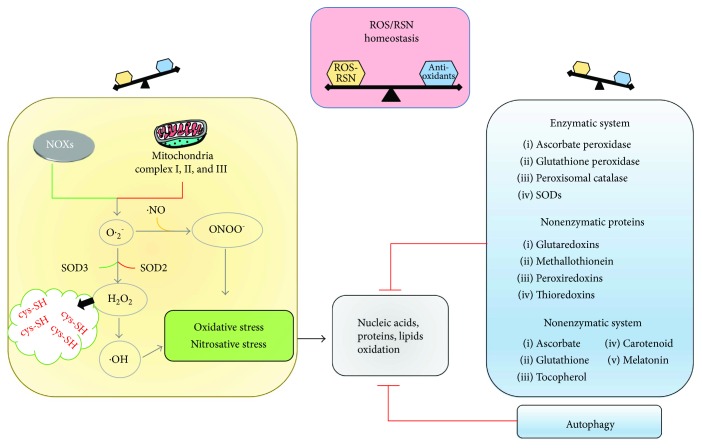
Reactive oxygen species (ROS) and reactive nitrogen species (RNS) balance is critical in maintaining cellular homeostasis. Excessive levels of ROS (O_2_⨪, ^•^OH, and H_2_O_2_) and/or RNS (ONOO^−^) affect the redox homeostasis, inducing oxidation of cellular nucleic acids, proteins, and lipids. The cells activate several antioxidant systems to maintain the intracellular redox equilibrium, including an enzymatic system (ascorbate peroxidase, glutathione peroxidase, peroxisomal catalase, and SODs) that works in concert with other nonenzymatic proteins (glutaredoxins, metallothionein, peroxiredoxins, and thioredoxins) and an nonenzymatic system (ascorbate, carotenoid, glutathione, melatonin, and tocopherol). In addition, autophagy is a very sensitive antioxidant system. NOXs = NADPH oxidases; cys-SH = cysteine-SH.

Living organisms have evolved enzymatic and nonenzymatic pathways that prevent oxidative damage to essential macromolecules, including proteins and nucleic acids. The pathways are modulated by several protein-based sensory, while regulatory modules ensure a rapid and appropriate response [39]. Peroxisomal catalase, SODs, glutathione peroxidase, and ascorbate peroxidase are antioxidant enzymes that remove O_2_⨪, H_2_O_2_, and peroxides in cell districts, acting as highly efficient antioxidant systems that protect cellular components by variable extent. The enzymes act in concert with other proteins as peroxiredoxins [40–43], thioredoxins (Trx) [44], glutaredoxins (Grx) [45], and metallothionein [46–48] and with low molecular weight, nonenzymatic antioxidants as ascorbate, glutathione [45, 49], tocopherol, carotenoid, and melatonin [50–53]. The oxidative signal is essentially reversed by two potent antioxidant systems the Trx/Trx reductase and Grx/Grx reductase, which reduce disulfides to free thiol groups at the expense of NADPH depletion. Antioxidant systems contribute to scavenge excessive ROS, thus finely controlling their levels and restoring the pools of reduced proteins and lipids (Figure 1).

### 2.3. ROS/RNS Effects

ROS/RNS exert different effects on the same targets, depending on cell type, with the exception of ^•^OH and ONOO− that are always associated to plain toxicity. The basal oxidation level that is necessary for correct cell viability and functions requires a redox homeostasis mechanism. Radical fluctuations are strictly controlled through their continuously balancing in, for instance, increased energetic demand, which intensifies electron flux through mitochondria, or aging, which decreases mitochondrial efficiency. Exogenous ROS/RNS sources, as oxidases and oxygenases, infrared and ultraviolet radiations, dietary nitrosamines, or chemotherapy agents [21], may contribute to redox homeostasis changes. Final effect of ROS/RNS, from now simply referred as ROS, is not exclusively determined by cellular concentration of each species but also by balance between different species, that is, H_2_O_2_ versus O2⨪. Indeed, O_2_⨪ from mitochondria may drive signaling pathways in cancer onset, development, and amplification. ROS trigger thiol oxidation, glutathionylation, nitrosylation, and carbonylation on specific proteins and enzymes, which consequently act as signal mediators in cell metabolism and signaling, even if the exact mechanisms have to be clarified [38, 54, 55]. Both cytosolic and nuclear proteins are ROS target containing ROS-sensitive cysteine residues that play regulatory rather than structural roles. These cysteines react as molecular switches that transduce redox signals, conferring redox activity to the proteins through their thiol groups. After undergoing oxidative modification and generation of S-hydroxylated derivatives, protein conformation/function is modified by reacting with other cysteines that generate either intra- or intermolecular disulfides, the last promoting complexes to conduct new functions. Redox-activated proteins act as intracellular redox sensors that allow for ROS properly adapting to their functions in the cellular redox equilibrium [21, 56]. Actually, these sensors result useful for studying pathogenesis and progression of multiple diseases [39, 55]. In particular, physiological trace levels of H_2_O_2_ act as both sensor and second messengers, being able to cross membranes, and induce specific signal transduction pathways in the cell [55]. ROS contribute to cell homeostasis as “second messengers” by modulating the activities of key regulatory molecules, including protein kinases, phosphatases, G proteins, and transcription factors. Periodic oscillations in the cell redox environment regulate cell cycle progression from quiescence (G0) to proliferation (G1, S, G2, and M) and back to quiescence, as a redox cycle. A loss in the redox control of cell cycle could lead to aberrant proliferation, a hallmark of various human pathologies [57]. ROS role is continuously delineated in a variety of physio-pathological conditions including cell growth, proliferation, differentiation, aging, senescence, and defense against infectious agents during inflammatory responses [58, 59].

### 2.4. Oxidative Stress

Excessive ROS (O_2_⨪, ^•^OH, and H_2_O_2_) or RNS (peroxynitrites and nitrogen oxides) and their reactive metabolites may be derived from imbalance between oxidant generation and removal by antioxidants that disrupts the redox homeostasis. The condition, named oxidative/nitrosative stress (OS/NOS, simply referred as OS), is potentially harmful because increasing levels of excessive radicals induce improper signaling or oxidation of the main essential cell molecules. Bases in nucleic acid, amino acid residues in proteins, and fatty acids in lipids show different susceptibility to OS that allows for a finely organized signaling system. OS consequences depend on cell type so that it is hard to clearly differentiate OS and redox signaling. Cellular OS level moderately overcoming cellular antioxidant level may provide selectivity for specifically targeted molecules and constitute a signaling mechanism, even after generating specific irreversible alterations of definite molecules [60–62]. Metabolic changes from cellular OS include (a) reduced ATP concentration, possibly caused by damaged mitochondria, (b) deactivated glyceraldehyde-3-phosphate dehydrogenase, which causes glycolysis inhibition, (c) increased catabolism of adenine nucleotides, (d) enhanced ATP consumption due to the active transport of oxidized glutathione, (e) increased cytoplasmic calcium concentration from deactivated calcium pumps, (f) cell membrane depolarization, possibly due to deactivation of K, Ca, and Na channels, resulting in increased cell membrane permeability, and (g) decreased glutathione level and ratio between reduced and oxidized glutathione. Another dangerous event is the generation of oxidized glutathione in various connections with xenobiotics, products of lipid peroxidation, or proteins present in the cell. Increased ATP consumption occurs in disposing such products outside the cell that also contributes to reductions in cellular glutathione [63, 64]. Generally, excessive ROS irreversibly damage structures of main macromolecules, membranes, and organelles and hamper signal mediators activity, thereby representing a primary damage source in biological systems. Irreversibly oxidized biomolecules are essentially cleared from cells through the autophagic process that is consequently considered a very sensitive antioxidant system. Autophagy is a converging point of different inputs and underlies cell responses to stressful conditions affecting cellular homeostasis, from biomolecules integrity to cell viability. OS acts as a vital stimulus to sustain autophagy, with ROS being one of the main signal messengers, thus autophagy and ROS coordinate to maintain cellular homeostasis [25, 65]. Although the mechanism by which ROS activates autophagy remains unclear, an essential autophagy-associated protein Atg4 has been shown to be under redox control. S-glutathionylation of the AMP-activated protein kinase AMPK may also contribute to its activation by H_2_O_2_ exposure, which allows for autophagy progression [28].

### 2.5. Oxidatively Damaged DNA

**Figure 2:**
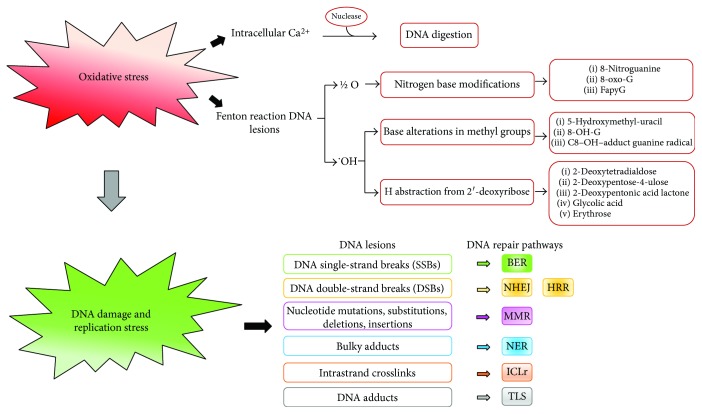
Oxidative stress (OS) causes DNA damage with consequent activation of DNA repair pathways. OS induces DNA damages by two major reactions: increase of the intracellular calcium levels, activating DNA digestion, and Fenton reaction. DNA damage triggers the main DNA repair pathways: BER: base excision repair; NHEJ: nonhomologous end joining; HRR: homologous recombination repair; MMR: mismatch repair; NER: nucleotide excision repair; ICL: intrastrand crosslink; TLS: translation synthesis.

The threat of cell molecule oxidation is a consequence of life in an oxygen-rich habitat that differently challenges molecule integrity and cell viability through the intermediate activity of homeostatic processes, mainly based on repair and degradation. Millions of DNA-damaging lesions occur every day in each cell of our bodies due to various stresses. Among which OS represents a major portion as it may induce approximately 10^4^ DNA lesions per cell of an organism per day. OS mediates the damage upon different insults such as ultraviolet, X- and *γ* radiations, pollutants, poisons, or endogenous disequilibria as metabolic imbalance that produce different and characteristic types of lesions. The lesions are particularly significant since they interfere with DNA replication that generates mutations, unless repaired in an error-free process, and alter the expression of protein, including transcriptional factors, and consequently signaling pathways and cellular behavior. Among endogenous and exogenous ROS/RNS, the O_2_⨪ is considered as a main candidate, responsible for genetic instability and malignant transformation. Oxidative DNA damage on bases of nucleic acid is repaired to a certain extent for maintaining the genome integrity, as evidenced by the DNA repair systems of the cell that are outlined below, but the damage may also escape the repair systems [66–68]. Both nuclear (nDNA) damage and mitochondrial DNA (mtDNA) damage are particularly significant as it can interfere with replication to generate lasting mutations [63, 69]. Although mitochondria possess quality control systems that include antioxidant enzymes, mtDNA is more susceptible to oxygen damage than nDNA, possibly due to (a) lack of nuclear proteins associated with mtDNA, which could protect it from damage, (b) less elaborate and efficacious repair system than nDNA repair machinery, and (c) the proximity of the respiratory chain where ROS/RNS are continuously generated. Two theories attempt to explain the cause of DNA damage: by the first, the damage results from a site-specific Fenton reaction, that is, the generation of a hydroxyl radical in the reaction of transition metal ions present in DNA with H_2_O_2_ and by the second theory, OS increases intracellular calcium concentration, which in turn activates nucleases digesting DNA [70, 71]. Reactions causing DNA damage and their breakdown products are a multitude exemplified by (a) lesions generated by ½ O as nitrogen modifications in DNA bases, in preference guanine, producing 8-oxo-7,8-dihydroguanine (8-oxoG) and (b) ^•^OH that adds double bonds and abstracts a H atom from methyl groups in DNA bases, producing molecules as 5-hydroxymethyl-uracil, C8-OH-adduct guanine radical, and 8-hydroxyguanine; ^•^OH targeting C atoms of DNA sugar moiety by abstracting a H from each C–H bond of 2′-deoxyribose, generating various molecules, as 2-deoxypentose-4-ulose, 2-deoxypentonic acid lactone, erythrose, 2-deoxytetradialdose, and glycolic acid [72, 73]. In complex, the above lesions cause base and tandem base modification, leading to DNA intrastrand crosslinks, DNA-protein crosslinks, mismatched pairs with damaged bases, stalled DNA replication forks, clustered lesions, and single- and double-strand breaks (SSB-DSB). SSB are due to modified DNA bases and abasic sites, apurinic/apyrimidinic sites, caused through purine and pyrimidine base damage as well as sugar moiety damage. SSB are the most common lesions that result from genotoxic insults by endogenous ROS [17]. Electrophilic molecules or intrinsic DNA instability or inhibition of topoisomerase, which traps cleaved DNA intermediates, may cause SSB. If not repaired, the damaged site may be bypassed by incorporating a mismatched deoxynucleotide during DNA replication. Many oxidative base lesions in DNA are mutagenic, provoking structural alterations, including transversions: G/T or A/C, or overall conformational changes, which may affect transcription and/or replication processes, leading to chromosome deletions with lethal effects. The most common base oxidations 8-oxoG mispair with adenine (8-oxo-G:A) and 5-hydroxycytosine with thymine thus causing replication stress. The accumulated lesions lead to pathological processes, as they result cytotoxic by causing mitochondrial dysfunction, mutagenic by causing genetic instability, and finally oncogenic. Also, the marker of inflammation 8-nitroguanine is considered a potential mutagenic [74]. A key cellular response to oxidative damage is the signaling through the JNK pathway. Depending on intensity and duration of the damage signal, this pathway leads to distinct alternative responses including DNA repair, antioxidant production, or cell death. When damage overcomes cell repairing systems, the damage signal (i.e., excessive ROS and products) drives JNK pathways toward proliferation arrest and or cell death that both play a fundamental role in cell homeostasis maintenance. These responses are highly relevant to cancer therapy, as tumors are often under OS that produces elevated JNK levels, and therapy often involves inducing DNA damage with the intention of driving cell death [75]. Generally, oxidative DNA damage is enhanced in tumors where increased metabolism, oncogenic signaling, and mitochondrial dysfunction produce 100-fold more 8-oxoG than in healthy tissues. Inflammation promotes carcinogenesis and generates ROS in tumor cell and its microenvironment that add to a high level of spontaneous DNA base deaminations. A consequent base mispairing is generated that is potentially mutagenic if not rapidly and efficiently repaired. Ever increasing ROS levels lead cells to death (apoptosis). This feature is exploited to exert therapeutic effect against cancer by therapy tailored to augment cellular ROS level. Oxidative damage is believed a potential double-edged sword in cancerogenesis and ROS-based anticancer. Although at low and moderate levels, ROS affect some of the most essential mechanisms of cell survival such as proliferation, angiogenesis, and tumor invasion, at higher levels, these agents can expose cells to detrimental consequences of OS including DNA damage and apoptosis that result in therapeutic effects on cancer. Understanding the new aspects on molecular mechanisms and signaling pathways modulating creation and therapy of cancers by ROS is critical in the development of therapeutic strategies for patients suffering from cancer [30, 76]. Antioxidants protect against genotoxic agents and alleviate their effects by decreasing primary DNA damage that reduces risk of mutation and tumor initiation. ROS enhances the localization of metallothionein (MT) in the nucleus where MT is more efficient than the reduced glutathione in protecting DNA from ROS attacks [76, 77]. The enzyme human mutT homolog detoxifies oxidized nucleotides thus potentially preventing 8-oxoG-induced mutations. It particularly eliminates 8-oxo-7,8-dihydro-2′-deoxyguanosine triphosphate that detoxifies oxidized nucleotides through its pyrophosphatase activity which is a potential target in cancer therapy [78, 79] (Figure 2).

### 2.6. DNA Repair in Oxidatively Damaged DNA

Cells have evolved several DNA repair pathways to deal with DNA damaged by OS that sense DNA lesions and process them into appropriate structures for DNA damage response (DDR) activation. DNA lesions and corresponding repair mechanisms have been reviewed by Curtin [17] and Chatterjee and Walker [80]. A part from the simplest form of DNA repair that is the direct reversal of the lesion, the cells are equipped with a variety of distinct, although partially compensatory, DNA repair mechanisms, each addressing a specific type of lesion. There are multiple types of DNA damage in humans as well as distinct but interrelated DNA repair mechanisms. Dysregulation of the mechanisms plays a key role in cell genomic instability. Among the repair pathways, tolerance mechanisms are also comprised as the translesion synthesis (TLS) that is composed by specialized DNA polymerases and regulatory proteins able to confer viability in the presence of unrepaired damage. Examples of the most common mechanisms to repair oxidatively damaged DNA regard the repair of modified bases by direct repair and base excision repair (BER) [81, 82], base mismatch repair by mismatch repair pathway, intrastrand crosslinks (ICL) by a complex repair that involves Fanconi anaemia pathway (FA), nucleotide excision repair (NER) [83, 84], TLS and homologous recombination (HR) [85], DNA-protein crosslinks by ICL repair and NER, stalled replication forks by HR, NER, and FA, single-strand breaks (SSB) by BER and HR, double strand breaks (DSB) [85, 86] by HR, and nonhomologous end joining (NHEJ) [87, 88]. The most deleterious lesions produced by many chemotherapeutic agents that block replication and transcription are represented by ICLs. NHEJ is thought to be the primary means of repair for therapeutically induced DSBs resulting from ROS-inducing anticancer treatments. Selective DNA repair inhibitors are considered efficacious in cancer therapy with minimal host toxicity [89–91] (Figure 2).

## 3. DNA Damage Response (DDR)

**Figure 3:**
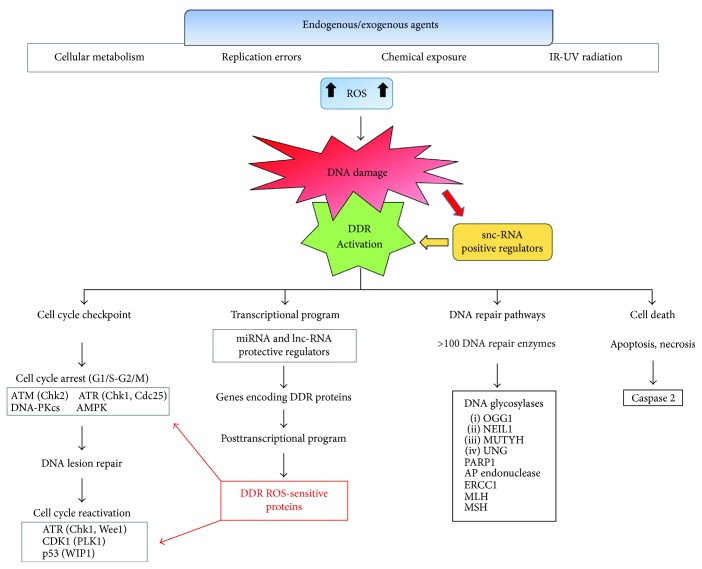
Reactive oxygen species (ROS) generated by endogenous and exogenous agents cause DNA damage and activation of DNA damage response (DDR). DDR activation arrests the cell cycle progression to repair DNA lesions and activate a program encoding ROS-sensitive proteins involved in DDR. ATM, ATR, DNA-PKs, AMPK, Chk1, and Chk2 represent the sensors and transducers that coordinate DDR. Their signals converge on effectors, as tumor suppressor p53, Cdc25 protein phosphatase, and WEE1 tyrosine kinase. DNA repair pathways occur by several DNA repair enzymes such as DNA glycosylases, PARP1, AP endonuclease, ERCC1, MLH, and MSH. DDR triggers apoptosis or necrosis when the DNA damage cannot be repaired. DDR-targeted proteins, whose inhibitors are currently in clinical trials, are indicated in bold. snc-RNAs = small noncoding RNAs; lnc-RNAs = long noncoding RNAs; ATM = ataxia telangiectasia-mutated protein; ATR = ATM- and Rad3-related; AMPK = AMP-activated protein kinase; CDK = cyclin-dependent kinase; DNA-PKcs = dependent protein kinase catalytic subunit; PLK1 = polo-like kinase 1; WIP1 = wild-type p53-induced protein 1; PARP = poly (ADP-ribose) polymerase; AP endonuclease = apurinic/apyrimidinic endonuclease; MLH = MutL homolog; MSH = MutS homolog.

The exogenous and endogenous insults upon human DNA result in accumulation of DNA damage that alters the chromatin environment besides increasing the mutagenic and immunogenic properties of the DNA [92–94]. The overall alterations possibly lead to physiological processes as aging and senescence or impact health and modulate disease states [95–97]. DNA damages induce and coordinate a complex signal-transduction network composed by several pathways, collectively named DNA damage response (DDR), that connects the DNA damage signaling to the cell cycle checkpoints maintaining cell homeostasis and functions while the damage is repaired. DDR prevents DNA duplication, cell division, and cell cycling, by arresting transcription process, to preserve genome stability and promote cell survival in front of both reparable or irreparable lesions [98]. If the damage is severe and irreparable, the cell cycle arrest is followed by cell death programs (apoptosis/necrosis) or senescence that eliminate damaged cells and avoid their multiplying. DDR initiates through phosphorylation-driven signaling cascades that sense the DNA damage, being regulated by mediators, and activate downstream effectors that finally determine the cell fate. It has been evidenced a set of 450 genes encoding proteins integral to DDR, among which a “core” group of proteins acts in different steps with some overlapping functions: (a) specialized “sensor proteins” detecting the damage; (b) transcription factors proceeding as “transducer proteins” upon their activation; and (c) “effector proteins” that are recruited by mediators. Other proteins organize and regulate a spectrum of processes that integrate DDR with the cell cycle progression allowing for the DNA repair [99, 100]. Each step in DDR is tightly regulated by reversible posttranslational modifications including phosphorylation, ADP-ribosylation, methylation, acetylation, ubiquitylation, sumoylation, and neddylation. Oxidatively induced DNA damage results in robust activation of three protein kinases that belong to the phosphatidylinositol-3-kinase- (PI-3-kinase-) related kinases of the PI-3-kinase/Akt pathway: (i) ataxia telangiectasia-mutated kinase (ATM); (ii) ATM- and Rad3-related kinase (ATR); and (iii) DNA-dependent protein kinase catalytic subunit (DNA-PKcs). The kinases are central components in DDR triggering and act together with the DNA repair machinery to maintain cell genome integrity [101–103]. ATM and ATR are activated through auto-phosphorylation as apical regulators of the response to DSBs and replication stress, respectively, with overlapping but nonredundant activities. A functional crosstalk between the major ATM/ATR pathways controls and coordinates DDR by affecting DNA replication, DNA repair, DNA recombination, mRNA transcription, and RNA processing, as well as protein metabolism and cell cycle. DNA-PKcs interacts with the DNA-binding Ku 70/80 heterodimer to originate the DNA-PK complex, a key regulator in NHEJ pathway that repairs the DSB damage. The first signal transduction wave is conducted by ATM/ATR phosphorylation that acts as DNA damage sensor and transducer. ATM activation is mediated through the Mre11-Rad50-NBS1 (MRN) complex that binds ATM through multiple protein-protein interactions, recruits ATM to DNA lesion as inactive dimer, and unwinds DNA ends to activate ATM. The complex MRN-ATM is located at the damaged DNA foci marked by histone *γ*-H2AX that is phosphorylated by the complex and regulates various downstream mediators to coordinate the DDR. Despite their distinctive individual activities, ATM, ATR, and DNA-PKcs share many overlapping substrates and roles in the regulation of the cell cycle checkpoints as primary or secondary responders to several DNA lesions. Upon their activation, ATM/ATR phosphorylate the checkpoint kinases CHK2 and CHK1, respectively, that acting as effector proteins, and phosphorylate the A, B, and C isoforms of the Cdc25s phosphatases. The phosphatases lead to inactivate cyclin-dependent kinases (CDK) and arrest cell cycle either at G1/S or G2/M transition, depending on which CDK is inhibited. CHK1 has a double role in CDK1 inactivation, by directly inhibiting Cdc25 and activating the tyrosine kinase Wee1, which specifically inhibits CDK1. Cdc25s control the cell cycle via specific checkpoints in physiological conditions as well as in response to DNA damage. These phosphatases transmit the damage signaling to effectors such as the tumor suppressor p53, a key molecule interconnecting DDR, cell cycle checkpoints, and cell fate decisions in the presence of genotoxic stress; p53 leads to cell cycle arrest or senescence or apoptosis depending on the damage extent and the cellular context. Inactivating mutations in TP53 gene and other genes involved in DDR potentiate cancer development and influence cancer cell sensitivity to anticancer treatments [21]. A novel genomic stress sensor in the DDR pathway is the AMP-activated protein kinase (AMPK) that is physically associated with the mitotic apparatus and participates in cytokinesis. AMPK has been previously known as a metabolic stress sensor, able to control cellular growth and mediate cell cycle checkpoints in cancer cells in response to low energy levels. AMPK is a key effector of the tumor suppressor liver kinase B1 (LKB1), which inhibits the cell growth mediator mammalian target of rapamycin (mTOR) and activates checkpoint mediators such as p53 and cyclin-dependent kinase inhibitors p21 (cip1) and p27 (kip1). Ionizing radiation and chemotherapy activate AMPK in cancer cells to mediate the signal transduction downstream of ATM that activates p53-p21 (cip1)/p27 (kip1) and inhibits mTOR. AMPK works as a convergence point of metabolic and genomic stress signals, which (i) controls the activity of growth mediators, (ii) propagates DDR, and (iii) mediates the antiproliferative effects of common cytotoxic cancer therapy such as radiation and chemotherapy. This highlights the importance of targeting AMPK with novel cancer therapeutics [104]. Also, it is worthwhile mentioning that the Wnt/beta-catenin signaling pathway, which is pivotal for modulating cell fate, proliferation, and apoptosis, can activate oxidatively induced DDR by regulating various proteins as histone *γ*-H2AX, p16INK4a, p53, and p21 [105]. Irreparable DNA lesions trigger elimination of damaged cells by apoptotic pathways like the autophagy form named “mitophagy” that leads to lysosomal degradation of damaged mitochondria [106, 107]. ATM links DDR to mitophagy induction by activating the LKB1/AMPK pathway, which in turn activates TSC2 by phosphorylation, thereby inhibiting mTORC1 and removing its inhibitory effect on mitophagy. Since autophagy contributes to clearing the cells of all the irreversibly oxidized biomolecules, it might be included both in the antioxidant system and the DNA damage repair system. Interestingly, it has been recently shown that some DNA repair enzymes can also activate and regulate the autophagy process [108, 109]. The indicated DDR pathways are involved in repairing oxidative DNA damage in healthy as well in cancerous cells, although following a different organization. Cancer cells frequently show several mutated molecules that lead to a reduced DDR activity thus facilitating the generation of further mutations and enhancing the cancer progression. Understanding the mechanism by which DDR is regulated under genotoxic stress should help improving the clinical outcomes [21] (Figure 3).

## 4. ROS-Sensitive Proteins Involved in DDR

Since when Rotman and Shiloh firstly proposed that ATM may act as a direct sensor and responder in cell OS and damage, accumulating body of studies has been reported. Attention is now focused on identifying the molecular contributions of ATM, ATR, and DNA-PKcs in the interplay between the DDR mechanism and the redox asset that comprehends the redox signaling, besides the oxidative DNA damage generated during the OS conditions [110, 111]. Indeed, several oxidative reactions contribute to redox signaling through finely modulating DDR at different levels, a part from causing oxidative genotoxic lesions. Interestingly, many proteins involved in DDR are endowed with a high number of cysteine residues (indicated in parenthesis) as exemplified by Chk1 (9), Wee1 kinase, a specific CDK1 inhibitor (12), Chk2 (13), Plk1 that allows cell cycle progression recovery after its arrest (13), and caspase 2 that is involved in apoptosis and is inhibited during G2 arrest by Chk1 (18). These ROS-sensitive proteins undergo modifications in their structure and function through cysteine residue oxidation and disulfide generation depending on the cellular ROS levels. In addition, some of these proteins activate pathways as p53 and p21 pathways, which finally lead to cell ROS level regulation. Through this loop mechanism, ROS contribute both to maintain the cell redox equilibrium and calibrate the DDR reactions [21, 112]. ATM is an OS-sensitive protein in which specific cysteine residues originate interprotein disulfides in human cells, upon being oxidized by ROS, thus resulting as an active homodimer. ATM is also activated through phosphorylation, as previously mentioned. The substrates phosphorylated by ATM are different following the MRN- or the OS-dependent activation, suggesting a different substrate specificity in the two conditions. While ATM phosphorylation initiates DDR in the nucleus, disulfide homodimer activates specific transcription factors in the cytosol, thereby leading to induction of antiapoptotic and prosurvival proteins. Through ATM activation, ROS lead to the recruitment of important proteins involved in DDR, including *γ*-H2AX histone and p53. The roles and localizations of ATM might be due to the presence of separate pools or ways of activation of ATM, or both the conditions that differently sense the cellular ROS levels. As very often OS and DNA damage overlap, the above conditions might collaborate in protecting the damaged cells from apoptosis while their DNA is repaired. It is difficult to discover the degree of overlap between substrates that are phosphorylated by ATM following DNA damage and substrates that are phosphorylated during OS, because the two ATM activities are usually exposed to both the conditions simultaneously. For instance, in anticancer treatments by ionizing radiations, both ROS production and DSB lesions are induced. The roughly 700 ATM targets that have been evidenced by a proteome analysis as probable targets in both DNA repair and oxidation pathways highlight a complicated interplay between oxidized ATM and DSB-activated ATM. The targets are mostly comprised of proteins involved in DNA replication, repair, and cell cycle control, as well as proteins affecting insulin signaling. This suggests that ATM may also function through regulation of metabolic signaling. In conditions that separate DNA from OS damage effects, only a subset of ATM targets that are usually phosphorylated in DDR is also phosphorylated in OS conditions. Now, ATM inhibitors of DDR mechanism are investigated as inhibitors of ATM redox functions. An ATM variant has been identified that is not activated by oxidation while is competent in DNA repair [81, 111, 113]. Interestingly, ROS may activate ATM independently of MRN, indicating that the OS-activated form has a primary role in redox sensing and signaling that may precede DNA damage and does not depend on it. Thus, MRN is not essential for ATM activation by OS, as the ATM pathway may also act separately from the DDR machinery. Evidences are known in which OS activation of ATM occurs in the absence of DNA damage, and OS inhibits ATM activation by MRN through disrupting the MRN-DNA complex [111]. This suggests that the only OS-activated ATM may operate under conditions of high ROS concentrations, playing a protective defense against the oxidative damage. Indeed, ATM deficiency is associated with elevated ROS, and ATM^−/−^ cells are more vulnerable to ROS-mediated OS, in comparison to normal cells [81]. Moreover, ATM inhibition enhances the sensitivity to the radiation therapy that generates ROS in cancer cells. The question is posed whether ATM may regulate global cellular responses to OS. Interestingly, ATM is activated in response to excessive ROS accumulation in vessels where it stimulates the neoangiogenesis of the endothelial cells by acting as a proangiogenic protein. The event is not due to defects in DDR pathway, since it is realized through a different signaling pathway from DDR, that is, the oxidative activation of the mitogen-activated p38*α* kinase. It is suggested that the pathological proliferating processes might require the ROS defensive system induced by OS activation of ATM. Targeting ATM might suppress tumor angiogenesis and enhance the effect of antitumor ROS-producing therapies. While loss of the activity of MRN-activated ATM may enhance the mutagenic effects of anticancer treatments and hamper the DDR barrier against tumorigenesis, the inhibition of the OS-activated ATM activity, which mediates oxidative defenses, might be efficacious in controlling malignant cell growth. The targeting of a cysteine residue that is crucial to the ATM activation by OS is believed a potential therapeutic strategy [21, 114]. Another important finding that demonstrates the interplay between ATM and OS is the ATM requirement for the ROS-mediated repression of mTORC1 [115, 116]. In response to elevated ROS, ATM activates the TSC2 tumor suppressor through the LKB1/AMPK metabolic pathway in the cytoplasm to repress mTORC1 and induce autophagy. The pathway acts as a node that integrates cell damage response with key pathways involved in metabolism, protein synthesis, and cell survival. The ATM interactor protein, ATMIN, is involved in the OS-induced ATM activity together with the SUMO (small ubiquitin-related modifier) enzymes as downstream ROS effectors, for cell survival under OS state. Replacement of a SUMO enzyme with a variant fails to maintain activated the ATM-DDR pathway normally induced by H_2_O_2._ The kinase ATR is also sensitive to modifications of the redox asset, comprising modified O_2_ supply and OS conditions. After being activated by replication inhibition during hypoxia conditions, ATR phosphorylates the Chk1 checkpoint signaling, p53, and histone *γ*-H2AX, activating the cell cycle arrest and the stabilization of stalled replication forks for allowing the subsequent reinitiation of the replication process [110, 112]. Similarly, the ATR-Chk1 checkpoint signaling is triggered by hyperoxic conditions in different in vitro models: human dermal HDF fibroblasts, human monocytes, lung adenocarcinoma cell line A549, and Xenopus egg extracts. In A549 cell line, the Chk1 checkpoint signaling is induced by ATR-mediated phosphorylation in an ATM-independent fashion, while in human monocytes, the ATM and ATR checkpoints are simultaneously activated by ROS-induced DNA damage. Moreover, the antioxidant lycopene, which is able to inhibit gastric pathologies related to oxidative DNA damage as 8-OH-G and DSBs, is also able to prevent ATM and ATR actions induced by ROS in gastric epithelial AGS cells. In summary, OS-activated ATR may precede OS-activated ATM operations showing that OS conditions affect the ATR and ATM interplay in the DNA repair pathways. How ATM and ATR checkpoint pathways regulate each other in response to OS remains to be elucidated [110, 112]. The DNA-PKcs mentioned as basic DDR actors are activated through their auto-phosphorylation by ROS accumulation and stimulate a series of reactions in signaling events typically triggered by OS, similarly to ATM. DNA-PKcs play a direct role in repairing oxidative DNA lesion through the BER repair pathways, although their mechanism in response to OS has to be clarified. Investigations are developing to determine roles and coordination between ATM and DNA-PKcs in OS signaling and oxidative DNA damage repair under both physiological and pathological conditions. This knowledge might offer new possibilities for the treatment of ROS-related diseases, including cancer [110, 111]. Among ROS-sensitive proteins in DDR, Cdc25 phosphatases (Cdc25s) and the checkpoint kinases CDKs are regulated by the intracellular redox milieu. The balance between kinase and phosphatase activity determines the strength of PI-3-kinase/Akt signal that may be modified through favoring kinase or phosphatase activity. Oxidations cooperate with DDR signals to activate kinases and inactivate phosphatases thus favoring the DNA repair. Cdc25s are direct OS targets since oxidation of cysteine residues in their active sites creates intramolecular disulfides causing the enzyme inactivation; thereby the cell cycle is arrested until favorable reducing conditions are restored. Cdc25s are inactivated by both oxidation and phospho-degradation. While oxidation is rapidly reverted, the phospho-degradation implies protein synthesis to be reverted. An oxidizing environment may increase the ratio between Cdc25 oxidation versus Cdc25 phospho-degradation, rendering the mitosis reenter easier and ultimately pushing cells toward proliferation. Cdc25s are overexpressed in tumor cells, which are generally endowed with a prooxidant environment, thus providing a mean for escape from the G2 arrest induced by the DNA damage [117, 118]. Another molecule that acts as OS sensor and cooperates with DDR is the tumor suppressor PTEN, protein tyrosine phosphatases, whose gene results one of the most frequently mutated genes in human cancers. PTEN exerts its tumor suppressor activity by regulating cell growth and survival through negative modulation of the P13-kinase/Akt signaling pathway. PTEN loss and/or inactivation causes abrogation of the checkpoint functions that control the cell cycle thus impairing DNA repair and genomic stability of the cells. Accumulation of DNA lesions and mutations causes tumor promotion. PTEN is inactivated by ROS through formation of an intramolecular disulfide bond between two cysteine residues that involves the protein active site. The inactivated PTEN induces a signal pathway that begins from Akt activation through phosphatidylinositol 3,4,5-trisphosphate, the PTEN physiological substrate, and terminates in the activation of antioxidant enzymes, possibly being an adaptive response to an oxidizing environment. The oxidized asset generally present in cancer cells may inactivate PTEN activity and, at the same time, allow for ROS acting as tumor promoters [118, 119]. A functional interplay between DDR pathways and DNA repair pathways occurs in response to OS, as DDR pathways not only arrest cell cycle progression but also directly participate in and facilitate DNA repair pathways. DNA repair proteins may sense oxidative DNA damage and process the damage into appropriate structures for DDR activation. In conclusion, DDR and redox environment exert a subtle reciprocal interaction, since enzymes participating to DDR are modulated by redox alterations and in turn act to modulate the redox equilibrium. A link between OS and PI-3-kinase/Akt pathway occurs in healthy as well as in cancer cells in which represents an advantage to the tumor survival [120, 121]. More intense investigations need to understand the interplay between ATM/ATR-mediated DDR pathways and DNA damage tolerance pathways in OS response. It is unclear how ATM-Chk2 and ATR-Chk1 pathways crosstalk with each other in response to OS. The new insights into ATM, ATR, and DNA-PKcs roles are a stimulus to identify points that may be redox regulated thus offering possibilities to treat ROS-related pathological conditions and diseases [25, 28].

## 5. Targeting DDR in Cancer Therapy

Anticancer treatments primarily target DNA damage, both directly and indirectly, in consideration of its role in malignant transformation and related consequences [15, 16]. The potential existence of distinct DNA damage thresholds at various stages of tumorigenesis and the role of the DDR pathway in human cancers are developed by Khanna [97]. DDR is rapidly induced, highly controlled, and regulated in cancer cells as in healthy cells suggesting the possibility of targeting definite DDR steps to hamper the cancer cell growth. The overall proteins of the DDR machinery may provide targetable intervention points for modulating DDR. It is worthwhile noticing that DDR protects and promotes cancer cell survival through restoring their reparable lesions, also when they are induced by DNA-targeted interventions. This event represents a main route to generate resistance against a genotoxic treatment. Dysregulation of DDR through missing or defective canonical pathways in the DNA repair mechanisms can lead to genomic instability that is a fundamental hallmark of cancer. Defective pathways may be eventually compensated for other DDR pathways generating a context, which highly favors cancer and resistance to genotoxic therapies [17]. Indeed, only cancerous tissues, but not healthy tissues, lack DDR components that render them dependent on the remaining compensatory DDR pathways. These compensatory pathways allow for cancer cells surviving in the ROS and replicative stress conditions that are present in cancer tissues. Since the event is cancer-specific, strategies that target compensatory DDR pathways may render a treatment-induced DNA damage more cytotoxic and preferentially eliminate cancer cells, while minimizing the impact on healthy cells. DDR inhibition has become an attractive therapeutic concept in cancer therapy, also for preventing or reversing the resistance to the anticancer treatments. [18, 122–126]. Indeed, dysregulated DDR is exploitable by both ordinary therapy and DDR inhibitors. While upregulated DDR confers resistance to DNA-damaging interventions and has to be inhibited to overcome such refractoriness, downregulated DDR makes tumor more susceptible to specific therapies and DDR inhibitors. In each single patient, the balance between the DNA damage induced by a genotoxic treatment and the consequent DDR is responsible for the effectiveness of the treatment. DNA repair-targeted therapies exploit DNA repair defects in cancer cells to generate their death resulting from simultaneous loss or inhibition of two critical functions. For example, cancer cells defective in one DNA repair pathway rely on alternate repair pathways, if inhibition of a second repair pathway occurs then results in cell death, an effect that selectively targets repair-deficient cancer cells [127–130]. This type of intervention, called synthetic lethality, is actually administered not only to selectively inhibit DDR in cancer cells with deficiencies in DNA repair pathway(s) but also to enhance chemotherapy and radiotherapy efficacy. A number of highly selective inhibitors that inhibit DNA repair pathways are in preclinical development, while others are clinically administered as DDR-targeted therapies in different stages of clinical evaluation. Poly (ADP-ribose) polymerase (PARP) inhibitors (PARPi) are the first clinically approved drugs designed to exploit synthetic lethality in cancer therapeutics that are clinically administered as DDR-targeted therapies to inhibit DNA repair pathways [131, 132]. PARPs are a family of DNA-dependent nuclear enzymes catalyzing the transfer of ADP-ribose moieties from cellular nicotinamide-adenine-dinucleotide to several proteins. This posttranslational modification is involved in cell response to DNA lesions, including DNA damage recognition, signaling, and repair as well as localized replication and transcriptional blockage, chromatin remodeling, and cell death induction. PARPs interact directly/indirectly, or via PARylation with oncogenic proteins and transcription factors, regulating their activity and modulating the carcinogenesis. For instance, PARPs regulate transcription factor-4 (ATF4) responsible for MAP kinase phosphatase-1 (MKP-1), which regulates MAP kinases. Very recent studies show that OS induces DNA breaks and PARP-1 activation causing mitochondrial ROS production and cell death. At the same time, PARPi reduce ROS-induced cell death, suppress mitochondrial ROS production, and protect mitochondrial membrane potential on an ATF4/MKP-1 dependent way, which inactivate JNK- and p38 MAP kinases. JNK is involved in the development of cancer stem cell, while JNK inhibition reduces the stem cell ability in tumor initiating. This could be a novel mechanism contributing to beneficial PARPi effects in combinatory cancer therapy with ROS-modulating drugs [133]. New therapeutic drugs such as PARPi are examples of DDR-targeted therapies that could potentially increase the DNA damage and replication stress imposed by platinum-based agents in tumor cells and provide therapeutic benefit for patients with advanced malignancies [134]. Indeed, many therapies are less effective by using one anticancer drug only, due to refractory properties and drug resistance in advanced cancers. A consensus is that anticancer drug cocktails might better control cancer progresses and metastasis than single drug therapeutics in clinical trials, but the complexity of drug combinations is still a challenge [135]. Investigation on cell cycle checkpoint signaling through ATM/ATR and pathways involved in cancer onset and progression has led to discover potent and selective ATM/ATR inhibitors that are actually in preclinical and clinical development, respectively. Experimental data have provided a strong rationale for administering ATR inhibitors (ATRi) since they cause synthetic lethality in cancers characterized by deficiency of certain DDR components. ATRi are assessed in clinical trials both as single agents and in synergy with various chemo- and radiotherapy therapies, including platinum, PARPi, and immune checkpoint inhibitors [17, 124, 126]. Preclinical data highlight the chromatin-bound phosphatase 2C isoform delta (WIP1) as potential target in human cancer. WIP1 is ubiquitously expressed at basal levels and is potentiated by p53. It acts as a strong negative regulator of p53 pathway thus forming a negative feedback loop that allows for terminating p53 response when DNA repair is completed. Genotoxic stress strongly induces WIP1 in cell lines in a p53-dependent manner (the WIP1 name refers to wild-type p53-induced protein 1). The substrate specificity of WIP1 matches the sites phosphorylated by ATM as p53, *γ*H2AX, and other DDR proteins. When overexpressed, WIP1 impairs p53 function and contributes to tumorigenesis, usually in combination with other oncogenes. WIP1 loss delays tumor development in mice, allows reactivation of p53 pathway, and inhibits proliferation in tumors endowed with p53. WIP1 is selectively inhibited by the small-molecule GSK2830371 that efficiently reactivates p53 pathway in various cancer types. In combination with DNA damage-inducing chemotherapy or with MDM2 antagonists (such as nutlin-3), WIP1 inhibition promotes cancer cell death or senescence, while healthy cells with basal WIP1 expression are relatively resistant to its inhibition [136].

## 6. Combinatory Anticancer Strategies Affecting ROS Levels

Most conventional chemo- and radio-therapeutic agents kill cancer cells in patients during cancer therapy by stimulating ROS generation as, at least, one part of their mechanisms of action [137]. ROS-inducing anticancer agents target mitochondria and enzymes in redox pathways resulting in OS conditions that lead to cancer cell death. The mode of cell death depends on the severity of the oxidative damage. Other major mechanisms of these anticancer agents inhibit or disable specific redox pathways and deplete reduced glutathione (GSH) [138]. It is believed that continuous investigations will allow the development of drug combinations for therapies better tailored to patients that cause fewer side effects and drug resistance [139]. Many cancer types may develop strong antioxidant mechanisms and maintain higher ROS levels than normal cells, but, at the same time, excessive OS levels may have tumor-suppressive effects [140]. This aspect offers an interesting therapeutic window because cancer cells might result more sensitive than normal cells to agents that cause further ROS accumulation. Examples of drugs with direct/indirect effects on ROS that are effective in cancer therapies are exemplified in the following sections in combination with DDR inhibitors, basing on the drug function in the cells. For better consulting of the drug combination, Table 1 shows combinatory therapies basing on the DDR target in the cells. Among the vast array of therapies, a single reference is reported either in brackets or as clinical trial number from https://clinicaltrials.gov/ (a database of privately and publicly funded clinical studies conducted on cancer patients).

### 6.1. DDR Inhibitors and Alkylating-Intercalating Drugs (Combinatory Therapies)

Therapies based on platinum coordination complexes (Pt-CC) as cisplatin (cDDP) [141–143], carboplatin (CarboPt) [144], and others, as well as therapies based on anthracyclines like doxorubicin, generate extremely high ROS levels, which may cause tumor cell death by apoptosis but also intolerable therapeutic side effects in the patients. cDDP is an alkylating DNA-damaging agent widely used as anticancer drug. It induces ROS via NADPH oxidase (NOX) and involves, inter alia, the activation of Akt/mTOR pathway, which is regulated by NOX-generated ROS [142, 145]. The combination of a large number of DDR inhibitors with Pt-CC impairs the defensive response of tumor cells against the Pt-CC-induced OS. For instance, the synergy between cDDP and PARP inhibitors (PARPi) that hampers the DNA damage repair may sensitize tumor cells to Pt-CC-induced OS. These combinatory therapies not only generate DNA damage foci and mitochondrial membrane damage in non-small cell lung cancer cells (NSCLC cell line) but also allow for reversing the resistance to the cDDP when it is administered as single agent. Olaparib or veliparib (PARPi) administration with Pt-CC is highly promising in different phases of clinical trials against some cancer types. Olaparib and cDDP administration in combination with radiation therapy (RT), which induces a substantial increase in ROS levels through NOXs activation [146], has been tested in advanced non-small cell lung cancer (NSCLC) (http://clinicaltrials.gov identifier: NCT01562210). In cancer treatments unsuitable for Pt-CC-based therapy as the oesophageal cancer, olaparib has been administered in combination with RT (http://clinicaltrials.gov identifier: NCT01460888). Veliparib and temozolomide [147] have been used to prevent repair processes following the ROS damage generated by CarboPt and paclitaxel [148] in metastatic breast cancer (http://clinicaltrials.gov identifier: NCT01506609). Rucaparib (PARPi) has been administered with CarboPt to advanced solid tumor patients (http://clinicaltrials.gov identifier: NCT01009190). A WEE kinase inhibitor, acting in the DDR mechanism, has amplified the oxidative damage induced by CarboPt, along with other cell killing actions, (http://clinicaltrials.gov identifier: NCT02087176). The compound MCI13E, which inhibits the replication protein A in the DDR mechanism, has also been tested preclinically in combination with cDDP [149]. A negative effect has been observed in the combinatory therapy between B02IR (RAD51 inhibitor) [150] with cDDP and mitomycin C [151], in which the OS caused by cDDP and mitomycin C results aggravated by B021R. Preclinical combinatory therapies between drug-inducing ROS and DDR inhibitors to overcome the resistance to Pt-drugs in solid tumors comprehend cDDP, NU-6027 (ATR inhibitor) [152], and hydroxyurea [153], among others, which is able to induce O_2_⨪ production. The DDR inhibitor VX-970 (ATR inhibitor) sensitizes cancer cells to the combination of CarboPt and the anticancer drug gemcitabine [154], which generates ROS by NOX and via NF-*κ*B activation in diverse cancer types (http://clinicaltrials.gov identifier: NCT02627443). Also, cDDP and gemcitabine have been administered with VX-970 against metastatic cancer (http://clinicaltrials.gov identifier: NCT02567409). Different DDR inhibitors, including ATM inhibitors, have been administered in combination with doxorubicin and other drugs to sensitize tumor cells to doxorubicin-induced OS and DNA damage [155, 156]. Doxorubicin induces oxygen-derived free radicals, particularly H_2_O_2_, through two main pathways: (i) a nonenzymatic pathway that utilizes iron and (ii) an enzymatic mechanism that involves the mitochondrial respiratory chain [157, 158]. Doxorubicin also inserts into DNA of replicating cells and inhibits topoisomerase II, causing double-strand DNA breaks and preventing DNA and RNA synthesis [159]. In conditions of DNA-PKcs inhibition, doxorubicin has been administered inside pegylated liposomes against advanced solid tumors (http://clinicaltrials.gov identifier: NCT02644278). Doxorubicin has also been combined with etoposide to concur with a dual inhibitor of DNA-PKs and PI-3K to kill tumor cells by causing, inter alia, mitochondria damage, GSH depletion, and ROS increase [160].

### 6.2. DDR Inhibitors and Folate Cycle Inhibitors (Combinatory Therapies)

Pemetrexed (PMX) and 5-FU are folate cycle inhibitors that also promote cytochrome c release from mitochondria and interfere with the electron transport chain, resulting in O_2_⨪ radical production and cell death [161, 162]. A DNA-PKcs inhibitor has been combined to 5-fluorouracil (5-FU) to improve the survival of patients with a form of metastatic pancreatic cancer that is refractory to the anticancer drug gemcitabine (http://clinicaltrials.gov identifier: NCT00045747). The cell reparatory response to the injury caused by PMX and cDDP is prevented by the contemporary administration of methoxyamine, an inhibitor of the DNA repairing AP endonuclease 1, thus resulting in a major efficacy of the therapy (http://clinicaltrials.gov identifier: NCT02535312). Prexasertib (LY2606368) inhibits the Chk1 enzyme involved in the DDR mechanism and has been tested in combination with 5-FU or PMX, or other drugs, against advanced or diffuse metastatic cancer (http://clinicaltrials.gov identifier: NCT02124148). PMX has been administered with the DDR inhibitor LY2603618 (acting against Chk1-Chk2) (http://clinicaltrial.gov identifier: NCT00988858) and in combination with cDDP to improve the survival of patients bearing advanced NSCLC (http://clinicaltrials.gov identifier: NCT01139775).

### 6.3. DDR Inhibitors, Immuno-Oncology, and Targeted Agents (Combinatory Therapies)

Immunotherapy is experiencing a growing interest as witnessed by the number of monoclonal antibodies that are administered in tumor patients as single agents or in combination with therapeutic interventions to prevent resistance to specific drugs. The monoclonal antibody cetuximab, which targets the epidermal growth factor (EGFR), has been combined with prexasertib (prevailing Chk1 inhibitor) or cDDP (http://clinicaltrials.gov identifier: NCT02555644) and the antifolates PMX or 5-FU, or other drugs (http://clinicaltrials.gov identifier: NCT02124148). Cetuximab downregulates the complex glutamine transporter ASCT2-EGFR in the cell membrane of non-small cell lung cancer cells (NSCLC cell lines). This causes that the glutamine necessary for the cellular GSH synthesis decreases, as well as the ROS reducing capacity of the cell. The consequent GSH reduction and OS trigger apoptosis independently of the EGFR-pathway downregulation [163, 164]. This increased sensitivity to OS has been exploited in association with the PARPi olaparib (http://clinicaltrials.gov identifier: NCT01758731). The monoclonal antibody bevacizumab, which causes cysteine and GSH level reduction and OS increase [165–168], has been administered together with the PARPi veliparib against metastatic colorectal cancer, and together with the PARPi niraparib against ovarian cancer (http://clinicaltrials.gov identifier: NCT02305758 and NCT02354131, resp.). The monoclonal antibody rituximab specifically binds to the CD20 antigen of B-cells, causing calcium influx into the cells and apoptotic signaling (reviewed in [167]). The antibody has been associated with veliparib against B-cell lymphoma [169]. In combination therapies, the proapoptotic process induced by rituximab often synergizes with the OS damage and O_2_^•−^ production caused by traditional anticancer interventions [170, 171]. Regarding targeted agents administered in combinatory strategies, tyrosine kinase inhibitors (TKIs) can affect the cell redox equilibrium in cancer cell lines and cancer tissues when administered in association with DDR inhibitors [172–174]. For instance, erlotinib enhances ROS production and induces ROS-mediated apoptosis in NSCLC A549 cell lines, via activation of the JNK pathway, leading to epidermal growth factor (EGFR) inhibition [173, 174]. Furthermore, erlotinib causes Nox4-induced H_2_O_2_ production in head and neck squamous cell cancer (HNSCC) cell lines [175]. The association between the TKIs erlotinib and gefitinib is approved for non-small cell lung cancer (NSCLC) treatment in tumors with specific EGFR mutations (10–15% of Caucasian patients). The TKi lapatinib is the only TKI approved for treating the human breast cancer subtype overexpressing the HER2 oncogene (20–30% of breast cancers). Lapatinib in combination with ABT-888 (PARPi) augments the cytotoxicity to ABT-888 resulting in efficacious synthetic lethality in HER2-positive breast cancer cells in vitro and in vivo [176]. Interestingly, the combination of lapatinib and the anticancer plant-derived berberine allows for reversing lapatinib resistance through the modulation of the ROS level [177]. In addition, a lapatinib analogue leads to ROS increase in the treatment of inflammatory breast cancer (reviewed in [167]). As a different example of targeted agents, bortezomib is the first ubiquitin-proteasome inhibitor approved as anticancer drug for human use [178]. This compound generates OS and aggravates the endoplasmic reticulum stress, causing apoptotic protein accumulation. Bortezomib has been proposed in association with ABT-888 (PARPi) [179–181].

### 6.4. DDR Inhibitors and Inhibitors of Topoisomerases I and II (Combinatory Therapies)

Inhibitors of topoisomerases I and II, such as topotecan and etoposide, cause single- and double-strand DNA breaks which inhibit DNA function and ultimately lead to cell death. These inhibitors induce OS essentially by increasing the endoplasmic reticulum stress and the oxidative status, as revealed by increased lipid and protein oxidation and decreased GSH and sulfhydryl levels in cancer lines [182, 183]. Evaluation of the chemotherapy improvement of topotecan action along with the drug VX-970 (ATR inhibitor) has been proposed (http://clinicaltrials.gov identifier: NCT02487095). In addition, the enhanced effectiveness of the combination between NU-7441 (DNA-PKcs inhibitor) [184] and etoposide [185], as well as KU-60648 (a dual inhibitor of DNA-PK and PI-3 K) with etoposide and doxorubicin, has been reported [160].

### 6.5. DDR Inhibitors and Direct Inhibitors of the Redox System (Combinatory Therapies)

It is well known that elevated GSH levels trigger chemo-resistance in cancer cells through different pathways: (i) direct interaction with drugs and ROS, (ii) prevention of damage of protein and DNA, and (iii) induction of DNA repair [186]. Several approaches for blocking GSH synthesis in cancer cells have been attempted, but, at the same time, cancer cells with high GSH content are more sensitive to drugs that affect GSH metabolism than normal cells. Buthionine sulphoximine (BSO) is the classical inhibitor of the rate-limiting enzyme in GSH synthesis that is used to increase cancer cell sensitivity to chemotherapeutics [187–189]. To this aim, the combination of 4-I-3 nitrobenzamide (PARPi) with BSO has been investigated in the E-ras 20 cancer cells that express the RAS oncogene, reporting enhanced cell killing [190]. Similarly, to GSH, changes to thioredoxin (Trx) metabolism are implicated in tumor cell resistance to chemotherapy. The gold compound auranofin (AF) is used as Trx inhibitor to induce OS, endoplasmic reticulum stress, and apoptosis in many tumor types, including cisplatin-resistant human ovarian cancer cells [191]. Cotreatment of mantle cell lymphoma (MCL) cells with AF and ABT-888 (PARPi) increases synergistically the apoptosis of ATM-proficient MCL cells, with increased *γ*-H2AX foci induction in the DNA and depletion of p-Chk1 (a downstream target of ATR signaling) [192].

## 7. Conclusions and Perspectives

The EU-ROS consortium comprising more than 140 members has worked for four years on the main topics of the redox biology and medicine. The results obtained highlight how synergistic approaches combining a variety of diverse and contrasting disciplines are needed in order to advance the knowledge of redox-associated diseases, including cancer [193]. ROS act as messengers that coordinate intracellular redox signaling in physiological and biological responses, as well as in tumorigenesis, suggesting that ROS-activated oncogenic pathways may also be regulated. Many strategies are under clinical investigations and trials that target the redox adaptation of cancer cells by redox-modulating interventions to both overcome drug resistance and eliminate selectivity cancer cells. Clinical efficacy of anticancer chemotherapies is dramatically hampered by drug resistance dependent on inherited traits, acquired defense against toxins, and adaptive mechanisms mounting in tumors. A heterogeneous cell population with distinct tumorigenic capabilities that complicate and limit the anticancer treatments may compose cancer tissues. Cancer plasticity leads to develop drug resistance by distinct mechanisms: (i) mutations in the target, (ii) reactivation of the targeted pathway, (iii) hyperactivation of alternative pathways, and (iv) cross-talk with the microenvironment. Molecular events leading to drug resistance are regulated by redox mechanisms suggesting redox-active drugs (antioxidants and prooxidants) or inhibitors of the inducible antioxidant defense as a novel approach to diminish the drug resistance. Repair and maintenance of cell genome stability show the cooperation between molecules that are essential to DDR and molecules essential to maintain the redox equilibrium. Ever increasing evidences highlight how the intricate molecular cross-talks between DDR and OS, generally indicated as OS-induced DDR pathways, can provide a useful insight into the drug discovery research aimed at counteracting cancer cell growth. Targeting DNA repair machinery has been a hot topic in anticancer therapy in the last decades. In fact, DDR inhibitors have been developed to increase the efficacy of conventional therapies and utilized in combinatory therapy with common cancer treatment, to overcome the therapeutic resistance to DNA-damaging chemotherapy and radiotherapy. This strategy can be used to selectively kill cancer cells with deficiencies in special DNA repair pathway(s) based on the concept of synthetic lethality. Although targeting DDR pathways is believed a promising therapy to fight solid and hematologic cancers, first early clinical trials with inhibitors in monotherapy have obtained scarce success. Currently, in order to optimize the application of these DDR inhibitors in the combinatory therapies overcoming resistance, massive array of preclinical and clinical trials are evaluating combinations of DDR inhibitors in targeted therapies. The best way to get a personalized medicine, matching the right treatment to the right patient, is based on identifying which patients have which DDR defect. The recent next generation sequencing (NGS) technology, which allows whole genomes to be sequenced in days, will be helpful to this strategy [194]. Today, an ever increasing range of available inhibitors targeting major DDR pathways allows for combining the inhibitors each other and with other targeted therapies and with treatments such as chemotherapy and radiotherapy, aiming at eliminating any escape road for cancer cells. In addition, there is an emerging impact of the promising immuno-oncology therapies as a new tumor treatment that might synergize with DDR inhibitions [http://clinicaltrials.gov identifier: NCT02484404] [195]. Recently, even the modulation of OS has been considered as a strategy that may affect some DDR pathways in human cancer and the responses to new anticancer therapies. For example, combinatory treatments between DDR inhibitors and agents that regulate indirectly or directly OS are very encouraging. The importance of this therapeutic strategy is supported by the results obtained from several ongoing preclinical and clinical studies exploiting combinations between DDR inhibitors and drugs that modify the ROS homeostasis (Table 1). The complexity of emerging categories of drugs targeting DDR and new strategies for integrating DNA repair-targeted therapies into clinical practice, including combination regimens, is a continuous challenge for both scientist and patients. Indeed, some caution are necessary for DNA repair-targeted agents as treatment with DNA repair inhibitors could increase mutation rates in malignant cells, leading to evolution of metastatic properties and/or drug resistance. Also, systemic DNA damage could increase the risk of secondary malignancies. While maximizing the cellular dependency on DDR inhibition often requires an oxidative DNA damage insult by chemotherapy or radiation, different levels of ROS and enzymes involved in their metabolism can participate in the DDR signaling. They can modulate the activity of key DDR enzymes and regulate the stringency of DDR by rendering the cancer cells more sensible to DDR inhibitors. Thus, lower doses of DDR target therapies might be administered to the patients. At the same time, the capacity of some chemotherapeutic agents to cause temporary perturbations in ROS levels can offer a therapeutic opportunity to both treat cancer and mitigate some toxic side effects of the chemotherapeutic agents. It is believed that the combination of ROS-affecting drugs with DDR inhibitors may help to define better-tailored therapies with fewer side effects and lower probabilities to promote drug resistance development.

## Figures and Tables

**Table 1:** DNA damage response (DDR) inhibitors in combination with ROS-inducing treatments for cancer therapy.

DDR target	DDR inhibitors	ROS-inducing treatments (direct/indirect mode of action)		References	Combinatory therapy Preclinical studies and clinical trials
PARP	Olaparib	Radiotherapy	OS increase by mitochondrial dysfunction	[146]	NCT01460888
		Cisplatin + Radiotherapy	ROS increase via NADPH oxidase	[141–143]	NCT01562210
			(♦)	(♦)	
		Cetuximab + Radiotherapy	Glutamine transport inhibition, GSH decrease	[163, 164]	NCT01758731
			(♦)	(♦)	
		Erlotinib	EGFR inhibition, ROS-mediated apoptosis	[173, 174]	[172]

PARP	Veliparib (ABT-888)	Temozolomide + Carboplatin + Paclitaxel	ROS increase, AKT–mTOR signaling disruption	[144]	NCT01506609
			ROS increase via NADPH oxidase	[147]	
			ROS induction	[148]	
		Bevacizumab	ROS and apoptosis increase	[165–167]	NCT02305758
		Rituximab	CD20 binding in B-lymphocytes, O_2_^−^ generation	[170, 171]	[169]
		Auranofin	H_2_O_2_ and ROS increase by thioredoxin reductase inhibition	[191]	[192]
		Bortezomib	ROS increase by ER stress	[178, 180, 181]	[179]
		Lapatinib	ROS increases	[176]	[176]
		Berberine	OS/NOS decrease	[177]	[177]

PARP	Rucaparib	Carboplatin	(♦)	(♦)	NCT01009190

PARP	Niraparib	Bevacizumab	Cysteine and GSH level reduction	[165–167]	NCT02354131
	4-Iodo-3-nitrobenzamide	Buthionine sulphoximine	Inhibition of glutamate–cysteine ligase complex in GSH synthesis	[187–189]	[190]

RPA	MCI13E	Cisplatin	(♦)	(♦)	[149]

RAD51	B02IR	Mitomycin C + Cisplatin	Stress-mediated ER cell apoptosis by ROS generation	[151]	[150]
			(♦)	(♦)	

APE-1	Methoxyamine	Pemetrexed + Cisplatin	Mitochondrial dysfunction, ROS increase	[161]	NCT02535312
			(♦)	(♦)	

ATM	KU-55933	Radiotherapy	(♦)	(♦)	[155]
		Doxorubicin + Radiotherapy	ROS increase by enzymatic/nonenzymatic pathways	[157]	[156]
			(♦)	(♦)	

ATR	NU-6027	Cisplatin	(♦)	(♦)	[152]
		Hydroxyurea	Increased O_2_^−^ production	[153]	[152]
	VX-970	Topotecan	ROS increase	[182]	NCT02487095
		Cisplatin + Gemcitabine	(♦)	(♦)	NCT02567409
			ROS increase, mitochondria alterations	[154]	
		Carboplatin + Gemcitabine	(♦)	(♦)	NCT02627443
			(♦)	(♦)	

DNA-PKcs	NU-7441	Etoposide	ROS increase, GSH depletion, mitochondrial alterations	[182, 183]	[185]
	KU-60648	Etoposide + Doxorubicin	(♦)	(♦)	[160]
			(♦)	(♦)	
	VX-984	Doxorubicin	(♦)	(♦)	NCT02644278
	UCN-01	5-Fluorouracile	Cellular O_2_^•−^ increase	(♦)	NCT00045747

Chk1/Chk2	LY2603618	Pemetrexed	(♦)	(♦)	NCT00988858
		Cisplatin + Pemetrexed	(♦)	(♦)	NCT01139775
			(♦)	(♦)	
	Prexasertib (LY2606368)	Cisplatin + Cetuximab + Pemetrexed + 5-Fluorouracile	(♦)	(♦)	NCT02124148
			(♦)	(♦)	
			(♦)	(♦)	
			(♦)	(♦)	
		Cisplatin + Radiotherapy + Cetuximab	(♦)	(♦)	NCT02555644
			(♦)	(♦)	
			(♦)	(♦)	

APE1 = AP endonuclease 1; ATM = ataxia telangiectasia-mutated protein; ATR = ATM- and Rad3-related; CHK = checkpoint kinase; DNA-PKcs = DNA-dependent protein kinase catalytic subunit; PARP = poly (ADPribose) polymerase; RPA = replication protein A. References in brackets; clinical trial identifiers (NCT). The effect of the single ROS-inducing drugs is indicated one time, and the following times is indicated with (♦).

## Abbreviations

AMPK: AMP-activated protein kinase
ATF4: Transcription factor-4
ATM: Ataxia telangiectasia-mutated protein
ATP: Adenosine triphosphate
ATR: ATM- and Rad3-related
BER: Base excision repair
CDK: Cyclin-dependent kinase
CHK1, CHK2: Checkpoint kinase 1, checkpoint kinase 2
cip1: Cyclin dependent kinase inhibitors p21
DDR: DNA damage response
DNA-PKcs: Dependent protein kinase catalytic subunit
DSB: Double-strand breaks
Grx: Glutaredoxins
HRR: Homologous recombination repair
ICL: Intrastrand crosslink
LKB1: Liver kinase B1
MMR: Mismatch repair
MRN: Mre11-Rad50-NBS1
mtDNA, nDNA: Mitochondrial DNA, nuclear DNA
NER: Nucleotide excision repair
NHEJ: Nonhomologous end joining
NOXs: NADPH oxidases
PARPi: Poly (ADP-ribose) polymerase inhibitor
PLK1: Polo-like kinase 1
PTEN: Phosphatase and tensin homolog
SOD2, SOD3: Superoxide dysmutase2, superoxide dysmutase3
SSB: Single-strand breaks
SUMO: Small ubiquitin-related modifier
TLS: Translation synthesis
Trx: Thioredoxins
WIP1: Wild-type p53-induced protein1
*γ*-H2AX: Gamma-histone2A.X.
